# Comparison of Elution Characteristics and Compressive Strength of Biantibiotic-Loaded PMMA Bone Cement for Spacers: Copal® Spacem with Gentamicin and Vancomycin versus Palacos® R+G with Vancomycin

**DOI:** 10.1155/2018/4323518

**Published:** 2018-10-16

**Authors:** Sebastian P. Boelch, Kilian Rueckl, Clara Fuchs, Martin Jordan, Markus Knauer, Andre Steinert, Maximilian Rudert, Martin Luedemann

**Affiliations:** ^1^Julius-Maximilians-University Wuerzburg, Department of Orthopaedic Surgery, Koenig-Ludwig-Haus, Brettreichstrasse 11, D-97074 Wuerzburg, Germany; ^2^Julius-Maximilians University Wuerzburg, Department of Trauma, Hand, Plastic and Reconstructive Surgery, University Hospital Wuerzburg, 6 Oberduerrbacher Strasse, D-97080 Wuerzburg, Germany; ^3^Hospital Agatharied, Department of Orthopaedic Surgery, Norbert-Kerkel Platz, 83734 Hausham, Germany

## Abstract

**Purpose:**

Copal® spacem is a new PMMA bone cement for fabricating spacers. This study compares elution of gentamicin, elution of vancomycin, and compressive strength of Copal® spacem and of Palacos® R+G at different vancomycin loadings in the powder of the cements. We hypothesized that antibiotic elution of Copal® spacem is superior at comparable compressive strength.

**Methods:**

Compression test specimens were fabricated using Copal® spacem manually loaded with 0.5 g gentamicin and additionally 2 g, 4 g, and 6 g of vancomycin per 40 g of cement powder (COP specimens) and using 0.5 g gentamicin premixed Palacos® R+G manually loaded with 2 g, 4 g, and 6 g of vancomycin per 40 g of cement powder (PAL specimens). These specimens were used for determination of gentamicin and vancomycin elution (in fetal calf serum, at 22°C) and for determination of compressive strength both prior and following the elution tests.

**Results:**

Cumulative gentamicin concentrations (p < 0.005) and gentamicin concentration after 28 days (p ≤ 0.043) were significantly lower for COP specimens compared to PAL specimens. Cumulative vancomycin concentrations were significantly higher (p ≤ 0.043) for COP specimens after the second day. Vancomycin concentrations after 28 days were not significantly higher for the Copal specimens loaded with 2 g and 4 g of vancomycin. Compressive strength was not significantly different between COP specimens and PAL specimens before elution tests. Compressive strength after the elution tests was significantly lower (p = 0.005) for COP specimens loaded with 2 g of vancomycin.

**Conclusion:**

We could not demonstrate consistent superior antibiotic elution from Copal® spacem compared to Palacos® R+G for fabricating gentamicin and vancomycin loaded spacers. The results do not favor Copal® spacem over Palacos® R+G for the use as a gentamicin and vancomycin biantibiotic-loaded spacer.

## 1. Introduction

Antibiotic-loaded bone cements are used to fabricate spacers during two-stage exchange of knee and hip prostheses for the treatment of periprosthetic joint infection (PJI) [1]. During stage-one operation, the infected prosthesis is removed and a poly(methyl methacrylate) (PMMA) bone cement spacer is temporarily implanted. The spacer works as a drug delivery system for antibiotics in order to achieve high local concentrations [2]. Additionally, it stabilizes the joint during mobilization and prevents soft tissue contraction [3]. Antibiotic loading of the bone cement can be performed right before the mixing procedure (manually loaded) or during the industrial production of the powder component (premixed). The spacer is molded after initiation of the polymerization of the cement by mixing its powder and liquid.

To date, some bone cement brands that are intended for the use for fixation of total joint replacements are also used to fabricate spacers, one example being Palacos® R+G [4, 5]. At the same time, cement modifications with improved antibiotic elution are tested [6]. Recently, a bone cement brand, designed specifically for fabricating spacers (Copal® spacem), was launched. The powder of this brand includes calcium carbonate particles, which serve as both contrast agent and biodegradable porogen. Antibiotics are not premixed into Copal® spacem, with the intention that an appropriate amount of pathogen-adjusted antibiotic will be mixed with the powder of the cement just before spacer fabrication and implantation. Bitsch et al. reported improved antibiotic elution characteristics of Copal® spacem when a single antibiotic was added [7].* In vitro* testing has shown that combination of antibiotics in a spacer can induce synergistic antibiotic elution [2] and superior antibacterial effects [8–10]. The combination of gentamicin and vancomycin ensures effective action against a broad range of PJI-causing pathogens [11]. Thus, there are reports of clinical use of spacers fabricated using manual loading of the gentamicin premixed powder of Palacos® R+G with vancomycin [1, 4].

In the present study, we compared antibiotic elution and compressive strength of Copal® spacem when gentamicin and vancomycin were added (COP specimens) to those properties for Palacos® R+G when vancomycin was added (PAL specimens). For COP, the amount of gentamicin added is the same as it is premixed in Palacos® R+G. For both cements, the antibiotic(s) were added to the powder using manual mixing. The investigation involved determination of the influence of vancomycin loading on the aforementioned cement properties and the compression tests were run both prior to and following the end of the elution tests. We hypothesized that gentamicin and vancomycin elution from COP specimens are significantly higher than from PAL specimens at comparable compressive strengths.

## 2. Materials and Methods

### 2.1. Specimen Preparation

Palacos® R+G (Heraeus Medical GMBH, Germany) contains premixed 0.8 g gentamicin sulphate (0.5 g active gentamicin). To produce equal gentamicin loading, 0.84 g gentamicin sulphate (0.5 g active gentamicin) (Caelo, Germany) was added to the powder of Copal® spacem (40 g) (Heraeus Medical GMBH, Germany). Then 2.05 g, 4.10 g, and 6.15 g vancomycin hydrochloride (Hikma Farmaceutica, Portugal) were added to the powder of Copal® spacem. The same amounts of vancomycin hydrochloride were added to the powder of Palacos® R+G. Thus, 6 cement formulations were used (Table 1).

Manual loading was performed following the recommendations by Kuhn et al. The antibiotics were thoroughly ground in a mortar and then the cement powder was successively added while stirring [4]. After that, the mixture was combined with the liquid of the cement, to produce a dough which then was poured into a mold to yield short, cylindrical specimens (diameter and height = 6 mm and 12 mm, respectively). These specimens were used for both the elution and the compression tests.

### 2.2. Elution Tests

The specimen was immersed in 1.5 ml fetal calf serum (FCS), in ambient laboratory conditions (temperature = 22 ± 1°C) for four weeks. FCS was exchanged every 24 hours. Samples of the eluate were taken every 24 hours for the first 7 days as well as after 14 and 28 days and stored at -20°C for determination of antibiotic concentration. Vancomycin concentrations were measured in a clinical analyzer (Hitachi Analyzer, Roche, Germany) with a homogene enzyme immunoassay (Online TDM Vancomycin Cobas, Roche (upper limit of measurement 50.0 *μ*g/ml and lower limit of measurement 5.0 *μ*g/ml with coefficient of variance 1.1%–4.9%)). Gentamicin concentrations were measured in the same way with the CEDIA® Gentamicin II Assay (Microgenics, Germany (upper limit of measurement 12.0 *μ*g/ml and lower limit of measurement 0.00 *μ*g/ml with coefficient of variance 3.0–6.2% for gentamicin)). If the concentration exceeded the upper limit of measurement, the eluate was diluted with FCS, the measurement was repeated, and the true concentration was calculated. For each of the formulations, 6 specimens were tested.

### 2.3. Compression Tests

The tests were conducted in accordance with the ISO 5883 [12] using a servohydraulic material testing machine (Z020, Zwick/Roell, Ulm, Germany), at a crosshead displacement of 10 mm/min. Tests were performed on two sets of specimens, prior to (n = 5) and immediately following the end of the elution test (n = 5).

### 2.4. Statistical Analysis

Results are presented as mean and 95% confidence intervals. The Kolmogorov-Smirnov-test was used to determine normal distribution of variables. Levene's test was used to test for equality of variances. T-tests for independent values were performed to identify significant differences in means. Significance was indicated if p < 0.05. All of these tests were performed using a commercially-available software package SPSS 24.0 (SPSS Inc., USA).

## 3. Results

**Figure 1:**
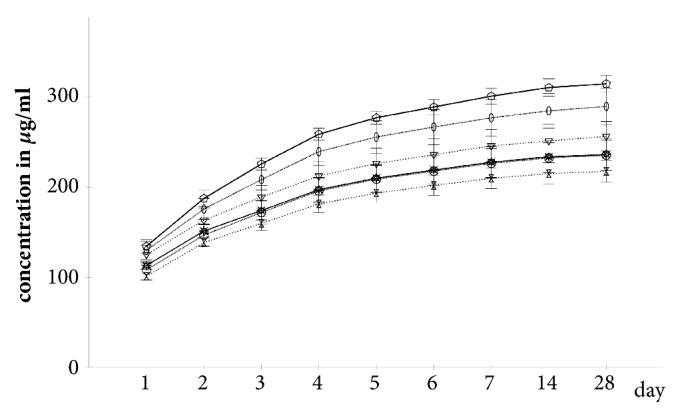
*Cumulative gentamicin concentrations in FCS sorted by specimen group*. Continuous line: vancomycin amount 6g; dotted line: vancomycin amount 4g; far dotted line: vancomycin amount 2g; asterisks: Cop6; pentagons: Pal6; circles: Cop4; ellipses: Pal4; double triangles: Cop2; triangles: Pal2; whiskers: 95% confidence intervals.

The COP specimens produced significantly lower cumulative gentamicin concentrations than the PAL specimens at each of the 9 time points (p ≤ 0.005) (Figure 1).

**Figure 2:**
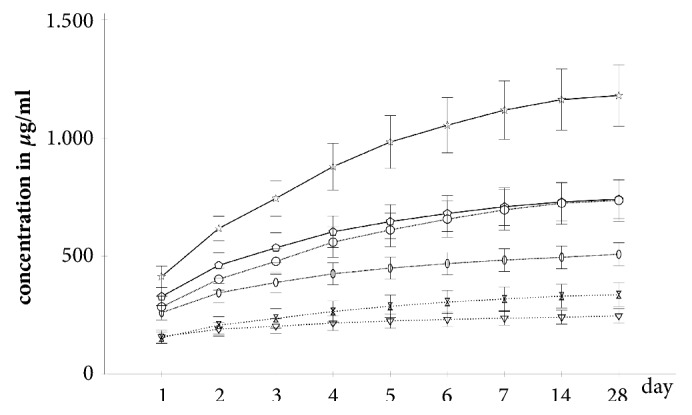
*Cumulative vancomycin concentrations in FCS sorted by specimen group*. Continuous line: vancomycin amount 6g; dotted line: vancomycin amount 4g; far dotted line: vancomycin amount 2g; asterisks: Cop6; pentagons: Pal6; circles: Cop4; ellipses: Pal4; double triangles: Cop2; triangles: Pal2; whiskers: 95% confidence intervals.

The Cop2 specimens produced significantly higher (p ≤ 0.043) cumulative vancomycin concentrations after day 2. For the Cop4 specimens significantly higher (p ≤ 0.035) cumulative vancomycin concentrations were measured after day 1 and for the Cop6 specimens at every measurement (p ≤ 0.004).  Figure 2 depicts the cumulative vancomycin concentrations

Long term elution is crucial for the treatment with spacers.  Table 2 depicts the determined antibiotic concentrations after 28 days.

Compressive strengths before elution tests were below the ISO 5883 recommended 70 MPa threshold, except for Pal4 (Table 3).

After the elution tests, the compressive strength of Cop2 was significantly lower (p = 0.005) than of Pal2. The compressive strength of the COP and the PAL specimens loaded with 4 g and 6 g of vancomycin underwent significant reduction (p ≤ 0.014) during antibiotic elution.

## 4. Discussion

Antibiotic-loaded PMMA bone cements are used as spacers to provide high local antibiotic concentrations and mechanical stability of the affected joint after removal of the infected prosthesis [2]. The bone cement Copal® spacem was recently introduced as a cement designed specifically for high antibiotic elution. However, data on antibiotic elution and mechanical properties of the Copal® spacem cement are lacking. The current study compared antibiotic elution and compressive strength of Copal® spacem and of Palacos® R+G as biantibiotic cements loaded with gentamicin and vancomycin.

For these biantibiotic formulations, we found lower gentamicin elution of the manually blended Copal® spacem compared to the gentamicin premixed Palacos® R+G. In contrast, Bitsch et al. found superior cumulative antibiotic elution from Copal® spacem in comparison to Palacos® R, when both these cements were manually monoantibiotic-loaded with gentamicin. In their study, vancomycin release displayed a slope of the cumulative antibiotic concentrations, which leveled earlier with decreasing amount of added vancomycin [7]. In accordance with these results, we found significant higher cumulative elutions but not higher vancomycin concentrations after 28 days for Cop2 and Cop4.

In the current investigation, manually gentamicin and vancomycin loaded Copal® spacem was compared to commercially available gentamicin premixed Palacos® R+G manually blended with vancomycin. Elution of Copal® spacem is enhanced by addition of calcium carbonate as a soluble porogen [7]. Such porogens enhance antibiotic elution by pore formation [6]. Although controversially discussed [13–15], premixed antibiotics are reported to be better eluted than manually loaded antibiotics [8, 16]. Ferraris et al. found larger inhibition zones around Palacos® R+G specimens compared to manually gentamicin loaded Palacos® R specimens, indicating higher antibiotic elution [8]. Comparable results were presented in the study by Lewis et al. demonstrating higher antibiotic elution from industrial loaded cements compared to manually loaded cements [17]. Thus, the elution enhancing effect of calcium carbonate in Copal® spacem did not compensate for the weaker elution of the manually loaded gentamicin compared to the premixed gentamicin. For the manually loaded vancomycin, the elution enhancing effect of calcium carbonate in Copal® spacem leads to higher cumulative antibiotic elution by increasing the initial burst release of vancomycin. However, high initial vancomycin elution goes along with enhanced vancomycin depletion. Consequently, the effect of improved vancomycin release by Copal® spacem fades by time in dependence of the amount of added antibiotic.

Compressive strength was reduced irrespectively of the amount of added antibiotic without significant differences between the COP and the PAL groups before the elution tests. After the elution tests, reduction of compressive strength was caused by void and crack formation due to antibiotic elution of the manually loaded antibiotics [18]. Beyond a critical antibiotic concentration, antibiotic elution leads to the development of a mechanically relevant percolation network causing significant reduction of compressive strength [16]. In our study, the specimens of both groups with 4 g and 6 g of vancomycin exceeded this critical antibiotic concentration. However, although the absolute amounts of antibiotics in the cement powders were the same for the corresponding groups, COP specimens had a higher proportion of manually added antibiotic than PAL specimens. The difference in compressive strength of the 2 g vancomycin groups shows that the critical antibiotic concentration of the manually added antibiotics for mechanically relevant percolation lies between Cop2 and Pal2.

The current study has a number of limitations. We examined antibiotic release by determination of concentrations, which does not allow conclusion on antimicrobial activity. Thus, we cannot state whether the higher antibiotic burst release of vancomycin from COP specimens is of advantage compared to the concentrations produced by PAL specimens. All measured concentrations exceeded the minimal inhibitory concentration of the most common pathogens for PJI, even after 28 days [19, 20]. After this time, antibiotic measurements were stopped, because concentrations fell below the lower limit of measurement for vancomycin. In contrast to antimicrobial testing, determination of concentrations was chosen for better comparability and the measurement method for its very low coefficient of variance. Furthermore, our study was limited to the antibiotics gentamicin and vancomycin. Other combinations need to be investigated, but the chosen combination is in clinical use [1, 4]. Finally, Copal® spacem has shown better wear behavior compared to Palacos® R* in vitro* [7]. This could reduce wear particle induced osteolysis, when used to fabricate articulating spacers. Copal® spacem might be advantageous for the treatment of PJI with a known pathogen as monoantibiotic-loaded spacer. However, if the pathogen is unknown, a biantibiotic-loaded spacer with vancomycin covering a broad spectrum of gram-positive and gentamicin covering a broad spectrum of gram-negative pathogens is warranted [11]. Our results are specifically relevant for this indication. For neither Copal® spacem nor Palacos® R+G, the addition of 4 g and 6 g of vancomycin can be recommended due to mechanical considerations [2, 21–23]. For the addition of 2 g of vancomycin, we found a higher initial burst release of vancomycin, but no significant difference of concentrations after 28 days. Additionally, significantly lower gentamicin concentrations were determined for Copal® spacem throughout the study. Based on these* in vitro* results, Copal® spacem is not of advantage for the use as a gentamicin and vancomycin biantibiotic-loaded, static spacer in comparison to Palacos® R+G.

## 5. Conclusion

Copal® spacem demonstrated inferior gentamicin elution. Cumulative vancomycin elution was significantly higher for all COP specimens, whereas vancomycin concentrations after 28 days showed no relevant differences. We could not demonstrate consistent superior antibiotic elution from Copal® spacem in comparison to Palacos® R+G as a biantibiotic gentamicin and vancomycin loaded cement. Thus, our results do not favor Copal® spacem over Palacos® R+G for gentamicin and vancomycin biantibiotic-loaded spacers.

## Figures and Tables

**Table 1:** Compositions of powder of the prepared specimen groups.

Specimen group	Active gentamicin amount in g	Active vancomycin amount in g
Pal2	0.5	2.0

Pal4	0.5	4.0

Pal6	0.5	6.0

Cop2	0.5	2.0

Cop4	0.5	4.0

Cop6	0.5	6.0

**Table 2:** Comparison of determined antibiotic concentrations after 28 days sorted by specimen group and measured antibiotic.

Specimen group by amount of added vancomycin in g	Measured antibiotic	Antibiotic concentration for COP mean in *μ*g/ml (CI 95%)	Antibiotic concentration for PAL mean in *μ*g/ml (CI95%)	p
2	Gentamicin	2.46 (2.03 – 2.91)	5.21 (2.62 – 7.80)	*0.041*

4	Gentamicin	2.97 (2.35 – 3.59)	4.93 (3.84 – 6.03)	*0.002*

6	Gentamicin	2.53 (2.02 – 3.03)	4.08 (2.43 – 5.73)	*0.043*

2	Vancomycin	5.52 (5.01 – 6.02)	5.52 (3.45 – 7.59)	1.000

4	Vancomycin	12.03 (11.19 – 12.88)	12.82 (6.80 – 18.84)	0.754

6	Vancomycin	17.73 (15.92 – 19.55)	11.45 (8.41 – 14.49)	*0.001*

**Table 3:** Comparison of compressive strengths before and after elution tests sorted by specimen group.

Specimen group	Compressive strength before elution mean MPa (95% CI)	Comparison of groups		Compressive strength after elution mean MPa (95% CI)	before vs. after elution p
		before elution tests p	after elution tests p		
Pal2	69.4 (66.8 – 72.0)	0.193	*0.005*	70.4 (67.3 – 73.2)	0.486
Cop2	66.9 (62.6 – 71.1)			63.9 (60.1 – 67.7)	0.173

Pal4	70.7 (65.5 – 76.0)	0.637	0.805	59.8 (57.5 – 62.1)	*0.001*
Cop4	69.5 (64.6 – 74.4)			60.1 (57.0 – 63.2)	*0.002*

Pal6	67.7 (58.2 – 77.1)	0.461	0.279	57.7 (53.8 – 61.6)	*0.014*
Cop6	65.1 (62.5 – 67.7)			55.5 (52.1 – 59.0)	*< 0.000*

## Data Availability

The data used to support the findings of this study are available from the corresponding author upon request.
